# Challenges and pitfalls in analyzing, reporting, and interpreting health effects related to occupational and environmental exposures

**DOI:** 10.3389/fepid.2026.1817746

**Published:** 2026-05-29

**Authors:** Glinda S. Cooper, Martha Powers, Krista Christensen, Suril S. Mehta, Ruth M. Lunn

**Affiliations:** 1 Retired, National Center for Environmental Assessment, Office of Research and Development, U.S. Environmental Protection Agency, Washington, DC, United States; 2 Radiation Protection Division, Office of Air and Radiation, U.S. Environmental Protection Agency, Washington, DC, United States; 3 Independent Researcher, Washington, DC, United States; 4 Division of Translational Toxicology, National Institute of Environmental Health Sciences, Research Triangle Park, NC, United States

**Keywords:** bias, environmental epidemiology, measurement error, misclassification, occupational epidemiology, risk assessment

## Abstract

Occupational and environmental epidemiology play a vital role in efforts to reduce preventable forms of disease and premature mortality worldwide by evaluating disease risk in relation to exposure sources, routes, and levels in a variety of industrial and community settings. There are considerable challenges in measuring these exposures due to wide variations in intensity, frequency, and duration. Because exposure levels and conditions often vary by site, comparing results across studies is also difficult. In this paper, we address these challenges and provide suggestions pertaining to the design, analysis, and reporting of individual studies as well as systemic reviews and meta-analyses. Key recommendations include reporting specific exposure levels within a study population and including quantitative bias assessment to address the impact of exposure measurement error on results within and across studies. These recommendations can improve the reporting quality and utility of occupational and environmental epidemiology, thereby strengthening its role in risk assessment.

## Introduction

Occupational and environmental epidemiology are among the oldest applications within epidemiology, dating back to the late 1700s and early 1800s with Percival Pott's observation of the risk of scrotal cancer among chimney sweeps (1) and John Snow's elucidation of the link between waterborne contamination and cholera (2). These fields continue to play a vital role in efforts to reduce preventable forms of disease and premature mortality. Key questions for meaningful risk reduction policies pertain to exposure sources, routes, and levels in diverse industrial and community settings. Occupational and environmental exposures can occur with varying degrees of intensity, frequency, and duration and often occur in combination with co-exposures that may create synergistic or antagonistic effects. These conditions create considerable challenges in accurately assessing, analyzing, and interpreting exposures.

**Figure 1:**
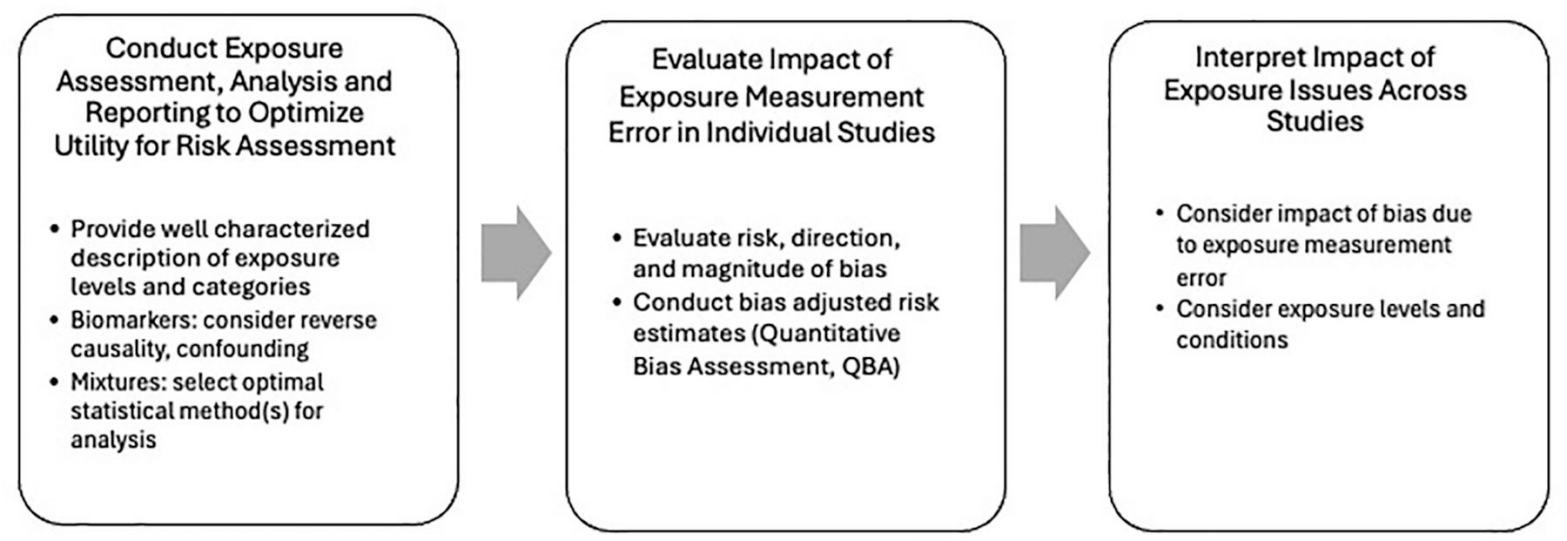
Addressing exposure measurement in individual studies and across collections of studies evaluating health effects of environmental and occupational exposures.

We address these challenges and provide suggestions for the design of individual studies and for methods to integrate results across studies (Figure 1). These recommendations aim to improve the use of epidemiology in risk assessments conducted by organizations to identify harmful effects of environmental and occupational exposures (hazard identification) and to characterize the likelihood of adverse effects at different exposure levels. Our focus is on exposure assessment, highlighting pitfalls in the choice and analysis of specific types of measurements and the importance of addressing error in exposure measurements. Because risk assessment involves the synthesis of evidence across studies, we also emphasize exposure-related issues that should be considered when evaluating and synthesizing a collection of studies.

The concepts of exposure and the methods that can be used to address it are included in comprehensive epidemiology curricula. Two recent publications evaluated the use of quantitative bias assessment (i.e., the type of approaches used and effect on results) among individual studies. They found the use of these methods to be relatively uncommon but increasing (3, 4). These evaluations included 258 (3) and 91 (4) studies, respectively, of which only 25 (11%) and 3 (3%) were environmental or occupational health studies. We also found that among the studies included in systematic reviews and risk assessments, quantitative bias assessment is seldom conducted. In addition, while most systematic reviews assess risk of bias, and some evaluate the direction of bias within or across studies (5, 6), quantitative bias analysis in published health hazard or risk assessments is extremely rare—if used at all.

## General exposure assessment issues

One issue in analyzing exposure measures based on environmental or biomarker samples is the shape of their distribution, which is often non-normal (e.g., left-skewed) or includes a large proportion of non-detectable values ,which may require use of non-parametric statistical tests or analysis of categories of exposure. Shaffer et al. provide suggestions for modeling and reporting of epidemiology results to facilitate their use in dose–response modeling and derivation of toxicity values (7). The distribution of exposures within a study, and within specific exposure categories, should be well characterized by providing measures of central tendency, minimum, maximum, and other variability parameters. Reporting exposure categories of “High,” “Medium,” and “Low” in a specific paper or analysis may provide a sense of the association between the exposure and a health outcome for a particular dataset, but such categorization is difficult to compare with other populations. Risk assessment requires examination of the potential risk at specific exposure levels, making clear delineation of exposure levels within each category essential.

It is also important to assess variability in exposure measures (e.g., by comparing a single spot urine sample to multiple pooled urine samples to reflect full-day excretion, or assessing variability in air concentrations of a pollutant over time). This type of analysis, or citations to such studies, should be included within a research report to allow consideration of quantitative bias assessment (8, 9).

## Biomarker exposure measures

Within population-based settings, biomarkers may not accurately represent a measure of exogenous exposure (10). In addition, biomarkers reflecting internal dose may be more susceptible to confounding by personal factors compared with proxy measures reflecting the external environment, resulting in challenges for study design, analysis, and interpretation (11). An additional issue concerns the relevance of the biomarker to the etiologically critical time window(s) of exposure. If an exposure biomarker is not collected within the relevant time window (e.g., collected concurrently with a prevalent condition), the potential for reverse causation should be considered when assessing health outcomes that may affect the pharmacokinetics (absorption, distribution, metabolism, or elimination) of the exposure. Reproductive and pregnancy-related outcomes, diabetes, adiposity, and thyroid diseases are among the examples of conditions that result in changes in hormone levels, blood volume, urine dilution, kidney function, or body burden (e.g., via elimination). Furthermore, treatment of prevalent disease (e.g., chemotherapeutics, surgery) may impact biochemical concentrations of exposures, leading to measurement error. Stratification or restriction based on relevant factors, such as parity or body mass index, can be used to address potential reverse causality. If associations persist or are not related to the factor, concern for reverse causality is lessened (12), which increases the utility of a study for hazard identification.

## Ambient environmental and occupational exposure measures

Available methods to estimate air quality for use in epidemiology and risk assessment include instrumentation (e.g., samplers, monitors, and satellites) and air pollution modeling approaches (e.g., land-use regression, dispersion, and chemical transport models) (13). It is challenging to obtain precise personal exposure data over time, and it is inherently difficult to estimate true personal exposure to ambient air pollution given the costs and infeasibility of monitoring individuals for multiple years. However, new air pollution monitoring paradigms, such as low-cost sensors and mobile monitoring frameworks, continue to emerge (14). In addition, the rise and rapid expansion of artificial intelligence methods for exposure assessment reflect the effectiveness and utility of these advances in air pollution monitoring and modeling (15).

Occupational epidemiology studies vary widely in the methods used to evaluate exposures, and the variability in sensitivity and accuracy affects their utility for hazard identification and risk assessment. For example, a silica pooled exposure–response analysis using quantitative job exposure matrices (JEMs) supported the International Agency for Research on Cancer (IARC) conclusion that inhaled crystalline silica is a human carcinogen and provided a basis for determining exposure limits needed to reduce cancer risk (16). The SYNERGY lung cancer study is another example of a particularly strong methodology using JEMs to integrate quantitative measurement data with prior occupational hygiene knowledge to build models incorporating personal exposure measurements, year of sampling, sampling duration, and an *a priori* exposure rating for each job (none, low, and high exposure). The model outcomes were then used to provide job-, region-, and time-specific exposure estimates for asbestos, respirable crystalline silica, nickel, chromium-VI, and polycyclic aromatic hydrocarbons (17).

## Issues relating to mixtures

Understanding the health effects of exposure to mixtures, which may include chemical and non-chemical stressors, is increasingly important in occupational and environmental epidemiology [see, e.g., Joubert et al (18)]. In toxicology studies, investigators may evaluate effects of a known mixture (i.e., whole-mixture approach) and compare these with effects from exposure to the individual components of the mixture. This approach can effectively identify when mixture components act in a synergistic or antagonist manner, versus when dose addition is an appropriate assumption. However, identification of whole-mixture effects is usually not possible in observational epidemiology studies given the myriad exposures encountered in everyday life through environmental media, diet, consumer products, and occupation. Instead, most epidemiology studies measure selected substances in environmental media or biological tissues. These exposure measures may be selected based on *a priori* hypotheses about causal associations with the health outcome of interest, but other considerations include convenience (e.g., availability in previously analyzed samples) and the ability to generate hypotheses for future study (e.g., exposome-wide association studies). Other grouping approaches may rely upon structural similarity (e.g., dioxin-like compounds) or co-occurrence (e.g., food packaging chemicals). Statistical approaches have been developed and applied to identify which mixture components are associated with health effects of interest, as demonstrated in the study by Zhan et al. examining associations between various per- and polyfluoroalkyl compounds and polycystic ovarian syndrome (19). Commonly implemented analyses include multivariate regression, penalization methods, and newer approaches such as Bayesian kernel machine regression (BKMR) and quantile G-computation. The choice of statistical methods depends on the research question being asked, but often will be influenced by the number of mixture components and the degree of correlation among them.

## Types of exposure measurement error

Measurement error is often divided into random and systematic types. Random error arises from chance processes, such as sampling variability—where a sample differs from its target population—and instrument-related fluctuations that produce inconsistent readings. Systematic error, in contrast, introduces a bias due to shortcomings in study design, data collection, or analysis. It can also take the form of differential misclassification of exposures or outcomes, particularly if certain demographic groups are more likely to inaccurately report exposure information.

Classical measurement error assumes that the factor has a true value that is measured with some amount of error; in other words, there is “noise” in the measured value (the expected value of the measured exposure is the true exposure). This concept is flipped for Berkson measurement error, where the measured value of the factor is fixed, and the true value fluctuates around that fixed value (the expected value of the true exposure is the measured exposure). This situation would occur, for example, when individuals are assigned group-level values, and true values vary randomly around that value.

## Assessing the potential for and impact of bias due to exposure measurement error

Exposure measurement error is a major challenge in epidemiology studies, and the utility of a study can be increased by using rigorous tools to assess the potential for error. Key elements include degree of concern (e.g., minimal, some, major), probability of bias (e.g., low, high), type of bias (e.g., differential, non-differential, Berkson, classical), direction of bias, and if possible, the magnitude of bias. Among the many tools available, the Report on Carcinogens Handbook (20) provides guidance tailored to different types of exposure assessments (e.g., questionnaire, biomonitoring). The Report considers the adequacy of proxies (such as biomarkers) for the exposure of interest as well as the quality of their measurement. In general, exposure mismeasurement or misclassification (for categorical variables) is a concern in nearly all environmental and occupational epidemiology studies. It is often non-differential, which on average biases risk estimates toward the null. However, there are a few notable exceptions: (1) non-differential but dependent misclassification of both exposure and outcome (e.g., self-reported exposure and outcome from the same questionnaire (21); (2) non-differential misclassification of multi-exposure categories (while each exposure classification can be toward or away from the null, the overall trend seen in the exposure–response relationship can be biased toward the null); and (3) Berkson bias, which results in imprecision and does not affect the magnitude of risk estimates for linear regression models, but may bias the effect measure in log-linear models (22).

The potential for bias is not the same as actual bias that impacts the results of a study. Exposure measurement error does not necessarily result in a large bias on findings, which is why bias impact assessment should be conducted to provide greater confidence in the interpretation of the results. The 2024 IARC report on statistical methods for evaluating bias impact in cancer epidemiology studies provides an excellent overview of this topic (23). With respect to non-differential and differential exposure mismeasurement, the report provides practical tools, including spreadsheets, code, and worked examples, to calculate bias-adjusted risk estimates for binary, continuous, and categorized exposure measures. An example of the application of these methods can be found in IARC's cancer hazard assessment of talc (24).

Bias-adjusted risk estimates are typically based on validation studies (e.g., assessing sensitivity and specificity), when available. If validation information is not available, a range of plausible risk estimates can be calculated using multidimensional analyses (combinations of assumed sensitivity or specificity) or probabilistic bias. The latter incorporates uncertainty in the bias parameters into the measures of association. For binary exposure, bias-adjusted risk estimates are calculated by adjusting the numbers of exposed cases and controls. For continuous data, risk ratios or hazard ratios for the outcome are adjusted using an attenuation factor, which is based on validation studies or a range of attenuation factors. One limitation of these methods is that they do not adjust for potential confounders. The IARC report also provides guidance for qualitative bias assessments, such as using negative control exposure to evaluate potential recall bias, and recommends visualizing exposure misclassification using directed acyclic graphs (DAGs) (23). We encourage the inclusion of these types of analyses in research reports.

### Examples of bias and bias assessment

Some analyses using the National Health and Nutrition Examination Survey (NHANES) biomarker data are based on blood samples that have been pooled across specific age-race-sex strata. When combined with sampling weights, these measures can be used to examine the distribution of the biomarker of interest in the population and make comparisons across defined strata. However, the inappropriate analysis of these pooled samples can result in a Berkson bias because the average value, rather than individual values, has been assigned to everyone in the strata. Unlike pooled samples within an individual, it is not valid to examine the association between these pooled measures and specific individual health outcomes among the participants who contributed to the pooled sample. The NHANES documentation cautions against doing this, noting that pooled-sample data files cannot be linked to other data files.

Examples addressing exposure error within environmental and occupational health research include studies of radon gas, an important source of ionizing radiation and contributor to the dose received by the general population (26). Some studies of radon and lung cancer have relied on surrogate measures of exposure such as type of home construction, presence or absence of a basement, and characteristics of local geology (27). There are multiple sources of error that can introduce uncertainty into estimates of long-term radon exposure, including temporal and spatial variations of radon concentration. A major uncertainty includes use of a 1-year radon measurement to approximate the average radon concentration in a home over a period of 20–30 years, as it assumes annual averages have remained stable over that period. Studies designed to assess year-to-year radon concentration variations within the home have been used to adjust the observed risk estimates between radon and lung cancer (28, 29), strengthening the utility of these studies for assessing risk associated with specific levels of exposure.

A recent study by Evangelopoulos et al. on air pollutants in relation to morbidity and mortality in London provides another example of approaches that can be used to address exposure measurement error (30). The authors applied data regression calibration and simulation extrapolation methods in conjunction with data from a separate study with personal exposure monitors to correct for measurement error from surrogate exposure measures. Both correction methods produced higher relative risk estimates compared to the uncorrected estimates.

In a study on occupational silica exposure and risk of systemic lupus erythematosus, Parks et al. demonstrated the impact of including detailed lifetime job histories—with information on specific tasks, industry, and occupation titles—to develop measures of exposure frequency, intensity, and probability (31). This more comprehensive exposure assessment method produced a relative risk estimate of 2.9 (95% CI 1.3–6.4) compared with an estimate of 0.4 (95% CI 0.2–0.9) based on standardized industry and occupation coding. This example demonstrates that surrogate exposures can be susceptible to measurement error when assessing individual exposure over time, which in turn can lead to biased effect estimates.

## Interpreting and comparing results across studies

Comparison of results across studies—for example, in the context of systematic review—requires assessment of the consistency and sources of variability in results. Because exposure measures may vary across studies, what may appear to be inconsistency may reflect true differences in exposure–response based on different exposure levels and so may strengthen, rather than weaken confidence in a set of studies. Thus, comparisons of results across studies should include careful consideration of the variability in exposure levels. The concept of study sensitivity—reflecting whether a study includes a sufficient exposure range and length of follow-up to observe a true effect—can be another useful component in evaluating studies for systematic reviews (32, 33).

In environmental and occupational studies, heterogeneity across studies can also be driven by differences in the quality of exposure assessment. We recommend a three-step process to evaluate the magnitude and direction of exposure measurement error bias. The first two steps, which can be addressed by the authors of specific studies (discussed earlier), involve assessing the potential for bias and the impact of bias in individual studies. The third step consists of assessing the impact of bias across studies in the context of systematic reviews or meta-analyses.

To evaluate the impact of bias across studies, stratification by quality and type of exposure assessment is recommended when synthesizing evidence using forest plots, subgroup meta-analyses, or meta-regression. For example, higher risk estimates were found for higher-quality exposure assessments in studies of pesticides and Parkinson disease, although exposure quality did not explain heterogeneity. Notably, all exposure assessments methods were associated with an increased risk of disease (34, 35). Our own unpublished analyses have also found in some, but not all reviews, higher risk estimates among studies for which there is less concern regarding the impact of exposure misclassification.

Unlike pharmacoepidemiology, where the concentration, timing, and administration of a dose to participants are known and tightly controlled, assessment methods of environmental exposures are often variable and subject to potential non-differential exposure measurement error. As such, the recategorization of measured data into levels, bins, dichotomous variables, scales, quantiles, and other methods may improve interpretability and provide more adequate exposure contrast. When considering the informativeness of disparate exposure assessments among studies, the adequacy of the exposure contrast (e.g., high exposure vs. low/no exposure) may be an important factor. Clear differentiation of exposure levels within a study minimizes mischaracterization of exposure assignments to participants, allows for a more accurate assessment of the exposure–response relationship, provides insight into sources of variation, and allows for a more valid effect estimate. In contrast, studies with a highly precise assessment of exposure concentrations but little differentiation between low and high exposure (e.g., low blood biomarker concentrations within a general population) may offer limited additional information despite a low likelihood of exposure measurement error (10).

Exposure measurement error in studies may not necessarily obscure associations with health outcomes when exposure levels are high. Despite less than precise exposure assessment measurements, the identification of individuals with high or very high exposure levels within a study population with an adequate exposure comparison group (i.e., lower or no exposure) mitigates bias toward the null from non-differential exposure misclassification, thereby increasing confidence in reported risk estimates. For example, when stratifying studies of trichloroethylene (TCE) by exposure level (i.e., high/very high, moderate, low), those with the highest TCE exposure concentrations were found to have the strongest association with kidney cancer, whereas studies with low TCE exposure saw no association with kidney cancer (5).

## Conclusions

Occupational and environmental epidemiology continue to play a critical role in identifying hazards associated with specific exposures and determining the levels of exposure needed to prevent disease. Occupational exposures to silica, metals, and other substances, and general-population exposures to air pollutants, endocrine disruptors, and other contaminants, remain a concern across countries at all levels of economic development (36–38). Researchers should be aware of the difficulties that can be encountered in designing and conducting individual epidemiology studies and systematic reviews or meta-analyses. Exposure categories should be linked to specific exposure levels for use in risk assessment. Biomarkers can be challenging to interpret, and the availability of samples should not be taken as a strong rationale for using them in an analysis. Associations observed between a biomarker measured concurrently with a health condition could be an artifact of reverse causality. This could happen, for example, when the health condition affects the pharmacokinetics (i.e., the absorption, distribution, metabolism, and excretion) of the exposure. The potential for exposure measurement error should be addressed in the design and during analysis, for example, by incorporating methods of quantitative bias assessment. The Report on Carcinogens Handbook (20) and IARC's report on statistical methods for evaluating bias impact (23) provide guidance and examples of qualitative and quantitative bias assessment. Statistical methods allowing for single and joint effects can be useful when analyzing health outcomes related to exposure mixtures. Exposure levels and measurement issues should be carefully considered when comparing results across studies, and occupational and environmental exposures included in systematic reviews and meta-analyses should be evaluated with methods designed specifically for these types of studies.

## Data Availability

The original contributions presented in the study are included in the article further inquiries can be directed to the corresponding author.
